# Editorial: Insights in Anti-doping Sciences: 2021

**DOI:** 10.3389/fspor.2023.1195599

**Published:** 2023-04-12

**Authors:** Raphael Faiss

**Affiliations:** REDs, Research & Expertise in AntiDoping Sciences, University of Lausanne, Lausanne, Switzerland

**Keywords:** anti-doping, rhEPO, hemoglobin, education, WADA, transfusion

**Editorial on the Research Topic** Insights in Anti-doping Sciences: 2021

The year 2021 may represent a turning point in anti-doping with the enforcement of a new World Anti-Doping Code after a thorough revision of the 2015 edition (1). This regulatory context also plays a pivotal role for research in anti-doping sciences and integrity with the new definition of substances of abuse, the notion of aggravating consequences, but also the expansion of laboratory reports for atypical findings beyond endogenous substances to consider putative contaminations. This research topic therefore aimed to outline multifaceted research perspectives of the latest scientific developments in recent years in the light of this new Code.

Practically one may first question the meaning of “clean” in anti-doping decision making. With the International Standard for Education (ISE) that came in force in 2021 and from a theoretical perspective, Petróczi & Boardley address “regulative, normative, and cognitive components of clean sport if we are to maximise its legitimacy” Petróczi and Boardley. Their review offers a pragmatic approach encouraging sensemaking in antidoping by conceptualizing doping as a sport-integrity issue.

From a more practical point of view, the concept of the indirect detection of doping by monitoring hematological and steroidal variations in athletes longitudinally (i.e., with the Athlete Biological Passport, ABP) still represents a useful strategy in the anti-doping toolbox. Recent research and discussion among anti-doping stakeholders in various workshop and conferences push towards testing and implementing new biomarkers for the ABP. However, hemoglobin concentration [(Hb)] as a primary biomarker for the hematological module of the ABP are influenced by plasma volume shifts. The perspective article presenting the future opportunities for the ABP Krumm et al. underlines major confounders as a starting point towards selection and validation of new approaches to rapidly reinforce the ABP. Krumm et al. interestingly highlight that research including the administration of doping substances result systematically in more atypical passport findings (ATPFs) in comparison to interventions including for example exercise training of hypoxic exposures while ATPFs are not excluded in the latter. Putting more weight on confounders to better target tests or interpret biological profile is therefore paramount.

Further, the comparative impact of either altitude exposure or recombinant human erythropoietin (rhEPO) injections on ABP parameters is evaluated. In their review, Saugy et al. reported that mean effects of rhEPO on blood parameters were greater than those induced by hypoxic exposure [1.7 times higher for (Hb) and reticulocytes percentage and 4 times higher for hemoglobin mass]. The interpretation of ABP profiles is hence facilitated by this identification of “temporal and quantitative evolution of blood parameters in connection with different hypoxic exposure doses, as well as different rhEPOs doses”.

With the evidence that the detection window and methods for EPO have improved, athletes regained interest in blood transfusions as an effective method to illegally boost their muscle oxygen convective transport. Homologous blood transfusion (HBT), i.e., with blood from a compatible donor, was widely used before it was forbidden in 1985 and athletes began to use rhEPO. Detecting autologous (reinjecting your own blood) however remains challenging with no internationally recognized method for its detection. In that context, an original research proposed a strategy to reduce false-negative results by improving the current antigen method Donati et al. minimize false negatives by expanding the antigen panel, selecting more accurately the gating area of the red blood cells and optimizing instrumental protocols. Their experimental approach was thus successful in samples simulating HBT between two compatible subjects.

The objective of this Research Topic is to provide well-documented insights by experts in the field to address the current challenges faced in the anti-doping efforts.

The body of evidence provided in this Research Topic supports the application of the updated 2021 World Anti-Doping Code as a robust basis to define priorities and strategies to tackle athletes trying to cheat. The included articles reflect research in this niche field ranging from perspectives to reviews with original research needing to answer pragmatically questions that arise in the daily implementation of the Code.

This Research Topic ultimately allows to collect and discuss existing evidence with practical proposals and empirical findings to strengthen the fight against doping.
